# A Flexible Copper Electrode Array for High-Density Surface Electromyography

**DOI:** 10.3390/bioengineering13040467

**Published:** 2026-04-16

**Authors:** Chaoxin Li, Chenghong Lu, Jiuqiang Li, Kai Guo

**Affiliations:** 1 Division of Life Sciences and Medicine, School of Biomedical Engineering (Suzhou), University of Science and Technology of China, Hefei 230022, China; zimuming236@gmail.com (C.L.); luch@sibet.ac.cn (C.L.); lijiuqiang@sibet.ac.cn (J.L.); 2 Suzhou Institute of Biomedical Engineering and Technology, Chinese Academy of Sciences, Suzhou 215163, China; 3 Jinan Guoke Medical Technology Development Co., Ltd., Jinan 250101, China; 4 Chongqing Guoke Medical Innovation Technology Development Co., Ltd., Chongqing 404100, China

**Keywords:** multi-channel sEMG sensors, serpentine interconnects, multi-site muscle monitoring

## Abstract

Precise monitoring of forearm muscle groups is crucial for decoding motor intentions in human–machine interfaces (HMIs) and rehabilitation. However, traditional surface electromyography (sEMG) electrodes face significant challenges in densely packed muscle regions with large skin deformations, leading to severe signal crosstalk and unstable contact. Here, we report a flexible, low-cost 16-channel copper electrode array system designed for the high-density monitoring of multiple forearm muscle activities. Through a facile fabrication process, rigid copper is transformed into a conformable sensing interface. The optimized serpentine interconnects endow the array with excellent stretchability and effectively isolate motion-induced stress, ensuring high-quality signal acquisition under complex deformations. The high-density 2 × 8 array enables the spatiotemporal mapping of distributed flexor and extensor muscle groups. Integrated with a customized wireless data acquisition system, the array successfully demonstrates real-time, multi-channel sEMG monitoring of various hand movements (e.g., fist clenching, wrist flexion/extension), clearly revealing specific muscle activation patterns. This low-cost, high-performance flexible sensor array provides a highly promising tool for complex gesture decoding, electromyographic imaging, and next-generation wearable HMIs.

## 1. Introduction

Surface electromyography (sEMG) is a fundamental non-invasive technique that records the physiological potentials during voluntary skeletal muscle contractions [1,2], encoding rich spatiotemporal information regarding muscle force and activation timing [3]. Consequently, sEMG sensing has been widely applied in advanced human–machine interfaces (HMIs) [4,5,6,7], robotic prosthesis control [8,9,10,11], and biomechanics [12,13,14]. Among various anatomical sites, the human forearm represents a highly critical yet challenging target [15,16,17]. It encapsulates densely packed flexor and extensor muscle groups that orchestrate the delicate, high-degree-of-freedom movements of the wrist and fingers [18]. Accurately decoding the dynamic activation patterns of these intricate hand gestures is essential for precise motor intention recognition [19]. This capability serves as the crucial technological foundation for post-stroke hand function rehabilitation monitoring, fine-grained neuromuscular assessment, and the control of next-generation rehabilitation robotics [20,21,22].

Despite its significance, the acquisition of high-fidelity sEMG signals from the forearm remains a significant challenge due to the inherent limitations of conventional electrode technologies. Traditional pre-gelled Ag/AgCl electrodes are typically rigid and bulky. This creates a severe mechanical mismatch with the soft, curved, and constantly deforming skin of the forearm during muscle contractions [23,24]. As muscles bulge and skin stretches during dynamic movements, these rigid sensors fail to maintain conformal contact, leading to significant motion artifacts and a drastic reduction in the signal-to-noise ratio (SNR) [25]. Furthermore, conventional sEMG acquisition often relies on sparse, discrete electrode configurations. In the anatomically dense forearm region, where numerous small muscles are packed closely together, these sparse sensors provide extremely limited spatial resolution. They are highly susceptible to signal crosstalk—a phenomenon where electrical potentials from adjacent muscles overlap—which obscures the specific motor information required for fine-grained gesture decoding [26,27,28]. Consequently, the lack of spatial granularity and the instability of the skin–electrode interface in traditional sensing methods hinder the precise monitoring of complex hand functions and neuromuscular assessments.

The rapid evolution of flexible electronics has significantly catalyzed the development of high-density surface electromyography (HD-sEMG) arrays, offering a transformative approach to muscular activity monitoring [29]. Unlike conventional sparse electrodes, these flexible HD-sEMG interfaces function as conformal “electronic skins” that seamlessly adapt to the intricate topography of the human forearm. This intimate skin–electrode coupling minimizes motion artifacts and reduces contact impedance, thereby substantially enhancing the SNR [30]. Current research emphasizes that such high-density configurations provide unprecedented spatiotemporal resolution, enabling the detailed mapping of muscle activation patterns rather than isolated point-source recordings [31]. This spatial granularity facilitates the visualization of motor unit action potential (MUAP) propagation, the estimation of muscle fiber conduction velocity, and the effective mitigation of signal crosstalk in anatomically dense muscular regions [32]. Consequently, these arrays have become indispensable for high-fidelity gesture recognition, fine-grained neuromuscular diagnostics, and quantitative motor function assessment in rehabilitation engineering [33]. However, the widespread clinical and industrial adoption of these high-performance interfaces remains constrained by their heavy reliance on expensive noble metals (e.g., Au, Pt) and sophisticated micro-fabrication protocols [34]. Furthermore, while recent advancements have explored alternative soft materials—such as silver nanowires, conductive polymers, or liquid metals—to achieve flexibility, these emerging systems often struggle with long-term electrical stability, complex material synthesis, and difficulties in standardizing interconnections with external rigid readout electronics [35,36]. Therefore, a critical gap remains in developing an HD-sEMG interface that bridges the compliance of soft bioelectronics with the reliability and scalability of standard industrial manufacturing.

To address this gap, copper emerges as a fundamentally transformative material for flexible sEMG arrays. Unlike laboratory-synthesized nanomaterials, copper offers unmatched electrical conductivity and is the ubiquitous standard in the printed circuit board (PCB) industry [37]. Utilizing copper intrinsically solves the persistent bottleneck of integrating soft sensors with rigid acquisition hardware. The primary challenge, however, lies in transforming inherently rigid copper foils into skin-conformal structures [38].

In this work, we present a cost-effective, high-density 16-channel flexible sEMG array fabricated from standard polyimide-backed copper (PI/Cu) laminates using a facile UV laser micromachining process. The array features a 2 × 8 configuration of miniaturized sensing pads interconnected by optimized serpentine traces. Notably, strategically designed circular structures are incorporated at the intersections of the serpentine lines to maximize the effective contact area, thereby ensuring reliable signal transduction and enhanced interfacial stability. This structural innovation, combined with the lightweight and self-adhesive nature of the sensor interface, enables conformal contact with the complex topography of the forearm while effectively isolating motion-induced mechanical artifacts. Integrated with a customized wireless data acquisition system, the 16-channel array facilitates simultaneous, high-fidelity capture of distributed muscle signals with an exceptional signal-to-noise ratio (SNR). Experimental results demonstrate that the proposed system can precisely map the spatiotemporal activation patterns of forearm muscle groups during various hand gestures.

## 2. Materials and Methods

### 2.1. Materials

Glass substrates were purchased from GuLuo^TM^, Luoyang, China. A PDMS kit (SYLGARDTM 184 Silicone Elastomer) was obtained from the Dow Chemical Company, Orlando, FL, USA. The copper/polimide film (19 μm) was supplied by DuPont^TM^, Wilmingtom, NC, USA. Ecoflex-0030 (Part A and B) and the release agent Ease Release^®^200 were provided by Smooth-On, Macungie, PA, USA. The water-soluble tape ASWT-2 was made available by Aqasol^TM^, Malaga, WA, Australia. The Silibione RT Gel4317 A/B was acquired from Elkem^TM^, Moon Township, PA, USA. The Anisotropic Conductive Film ANISOLM^®^ AC-2056R was procured from Hitachi^TM^, Tokyo, Japan. The conductive silver paste was obtained from SINWE, Shenzhen, China. Other chemical agents were ordered from Alfa Aesar, Waltham, MA, USA. All electronic components were obtained from Mouser^TM^, Mansfield, TX, USA.

### 2.2. Fabrication of the Electrode Array

#### 2.2.1. Sensor Patterning

The PDMS prepolymer and curing agent were thoroughly mixed at a mass ratio of 15:1 and degassed, followed by pouring onto a clean 150 mm × 150 mm glass plate. A uniform film was obtained via spin-coating (500 rpm, 40 s) and curing on a hot plate at 90 °C for 15 min to prepare a sacrificial layer. After cooling, PI/Cu film was attached to the PDMS surface, secured by interfacial adhesion. The geometric patterns of the electrodes and sensors were designed using AutoCAD software (Version 2025; Autodesk, Inc., San Francisco, CA, USA). Subsequently, a 355 nm ultraviolet laser cutting system (LM-UV-3, Delong corporation, Suzhou, China) was employed to create designed patterns. The laser parameters were set as follows: pulse frequency of 50 kHz, scanning speed of 300 mm/s, and 9 repetitions per path to ensure complete cutting. The cutting path was designed to remove connections around the pattern periphery, keeping the to-be-removed material interconnected. Complete removal via a simple manual peeling operation yielded the target sensor structure.

#### 2.2.2. Preparation of Flexible Substrates

First, double-sided tape was adhered to a clean 150 mm × 150 mm glass carrier. Next, a matching double-sided release film was attached with its light release side facing the double-sided tape to fix the film in place and facilitate subsequent overall peeling. On the heavy release side of the film, the elastomer support layer was prepared first: Ecoflex-0030 parts A and B were mixed at a 1:1 mass ratio, degassed, poured onto the surface, spin-coated (500 rpm, 40 s), and cured at room temperature for 1 h. Once the Ecoflex layer was fully cured, the adhesive layer was prepared on top: Silbione RT Gel 4317 parts A and B were mixed at a 1:1 mass ratio, spin-coated on top of the Ecoflex layer (500 rpm, 40 s), and cured on a hot plate at 90 °C for 15 min. This yielded a composite flexible substrate consisting of an Ecoflex-0030 support layer and a Gel 4317 adhesive layer.

#### 2.2.3. Transfer and Integration

First, the patterned PI/Cu sensor was retrieved from the PDMS sacrificial layer using water-soluble tape and transferred onto the pre-fabricated bilayer flexible substrate. The tape backing was subsequently dissolved with deionized (DI) water, followed by air-drying. For top encapsulation, a customized mask was employed to shield the skin-contact electrode sites and the FFC connection pads. A final layer of Gel 4317 (1:1 mass ratio) was then spin-coated (500 rpm, 40 s) and cured. Upon removal of the mask, the exposed electrode regions were defined and ready for signal acquisition and external interconnection. Finally, the release film was peeled from the double-sided tape on the glass carrier, yielding a standalone, flexible sensing system.

### 2.3. Integration of the Electrode Array with the Recording Module

A thermal bonding process based on conductive silver paste was employed to achieve reliable signal transmission between the flexible sensing system and external readout circuits. First, conductive silver paste was selectively applied to the contact pin side of the Flexible Flat Cable (FFC, EasyEDA, China) using a customized mask. Subsequently, the FFC pins coated with silver paste were precisely aligned and laminated onto the prepared copper electrode connection pads on the flexible sensor, operated under a microscope. After applying appropriate contact pressure, the interconnection area was subjected to localized heating (150 °C, 10 min) to promote full curing and sintering of the silver paste. This process established a robust low-impedance electrical pathway and achieved mechanical anchoring between the FFC and the flexible substrate, ensuring the stability of the interconnection interface under dynamic stretching.

## 3. Results

### 3.1. Design of the Flexible Copper Electrode Array

**Figure 1:**
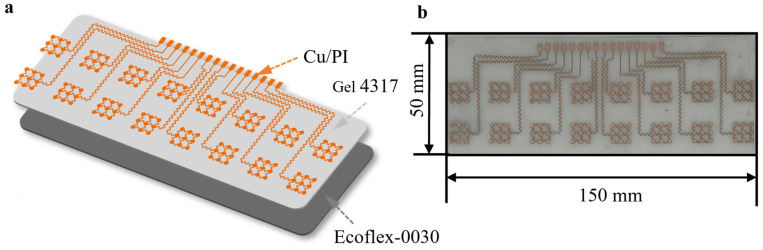
Overview of the flexible copper electrode array. (**a**) A schematic explosion view of the flexible copper electrode array illustrating the layered structure of electrodes and flexible substrates. (**b**) An optical image showing the overall dimensions of the flexible copper electrode array.

**Figure 2:**
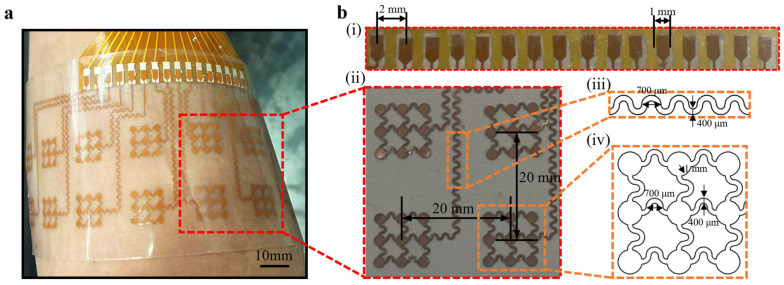
The detailed dimensioning scheme of the flexible copper electrode array. (**a**) A demonstration of the flexible copper electrode array conformally attached to the skin, highlighting its mechanical flexibility. (**b**) Detailed geometric dimensions of the flexible copper electrode array components: (i) FFC connection pads, (ii) electrode pitch and arrangement, (iii) the parameters of the serpentine interconnects, and (iv) the dimensions of the sensing electrode pads with circular intersection structures.

**Figure 3:**
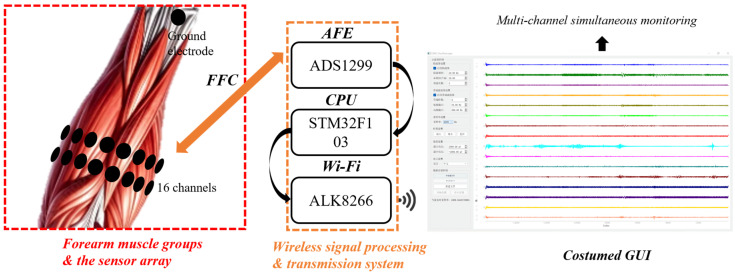
Overview of the wireless data acquisition and transmission system, illustrating the process from the signal recording of forearm muscle groups to real-time visualization on a customized GUI.

The primary objective of this study is to develop a high-density, conformable, and cost-effective sensing interface for multi-channel surface electromyography (sEMG) monitoring. The design logic of the flexible copper electrode array is systematically illustrated in Figure 1, Figure 2 and Figure 3, spanning from material selection and structural engineering to system integration.

As depicted in the exploded view in Figure 1, the array adopts a multi-layered hybrid structure to balance electrical performance with mechanical compliance. The core conductive layer is composed of a polyimide-backed copper (PI/Cu) laminate. We specifically optimized the thickness of the copper layer to 9 μm and the PI layer to 10 μm. The 9 μm copper provides a negligible sheet resistance, ensuring high-fidelity signal transmission without significant attenuation. Simultaneously, the 10 μm PI serves as an ultra-thin insulating support that maintains structural integrity while minimizing bending stiffness. For the substrate and encapsulation, a combination of Ecoflex-0030 and Gel 4317 was employed. Ecoflex-0030 was selected for its exceptionally low Young’s modulus (~30 kPa), which closely matches the mechanical properties of human skin, providing a soft and compliant base. Gel 4317 (1:1 mass ratio) was integrated due to its dual functionality: it acts as a breathable, biocompatible encapsulation layer and provides reliable self-adhesion. This tacky interface stabilizes the skin–electrode contact, effectively suppressing the relative sliding that typically generates motion artifacts.

The overall dimensions of the array were determined based on human forearm anthropometry and physiological requirements. As shown in Figure 1b, the array measures 150 mm × 50 mm. This length was strategically chosen based on average forearm circumference measurements (typically ranging from 23 to 28 cm in adults) to ensure that the array can wrap around approximately 50–60% of the forearm circumference. This extensive coverage allows for the simultaneous monitoring of both the flexor and extensor muscle groups, which is essential for decoding multi-degree-of-freedom hand and wrist movements.

The practical application of the array is demonstrated in Figure 2a, where it is conformally attached to the forearm. The ultra-thin nature of the PI/Cu layer (19 μm) combined with the soft Ecoflex substrate allows the device to seamlessly adapt to the complex, non-developable topography of the human limb. This conformal contact is critical for high-quality sEMG acquisition, as it eliminates air gaps and maintains a stable electrochemical interface even during vigorous muscular contractions or limb movements, thereby ensuring a high SNR.

The detailed geometric parameters of the flexible copper electrode array are systematically presented in Figure 2b. First, the connection terminal features a 2 mm width and a 1 mm pitch (Figure 2b(i)) to facilitate stable electrical coupling for multi-channel data acquisition. The sensing nodes are arranged in a 2 × 8 grid with an inter-electrode distance of 20 mm (Figure 2b(ii)), a pitch optimized to balance high spatial resolution with the mitigation of signal crosstalk between adjacent muscle fibers. To accommodate significant skin deformation, these nodes are interconnected by serpentine traces with a 700 μm amplitude and 400 μm width (Figure 2b(iii)), which function as micro-springs to ensure exceptional mechanical flexibility. Finally, circular structures are strategically integrated at the intersections of the serpentine lines (Figure 2b(iv)) to maximize the effective skin-contact area for high-fidelity signal transduction while effectively redistributing mechanical stress during movement.

Finally, the integrated data transmission and processing workflow is summarized in Figure 3. The flexible copper electrode array captures distributed sEMG signals from the forearm muscle groups, which are then routed via a Flexible Flat Cable (FFC) to a customized wireless acquisition system. The hardware utilizes a high-precision Analog Front-End (ADS1299) for low-noise amplification, an STM32F103 CPU for high-speed data processing, and an ALK8266 Wi-Fi module for stable wireless transmission. The signals are ultimately visualized on a customized Graphical User Interface (GUI) in real-time. This holistic system architecture enables the simultaneous monitoring of multi-channel muscle activation patterns, providing a robust platform for motor intention recognition and rehabilitation assessment.

### 3.2. Fabrication of the Flexible Copper Electrode Array

**Figure 4:**
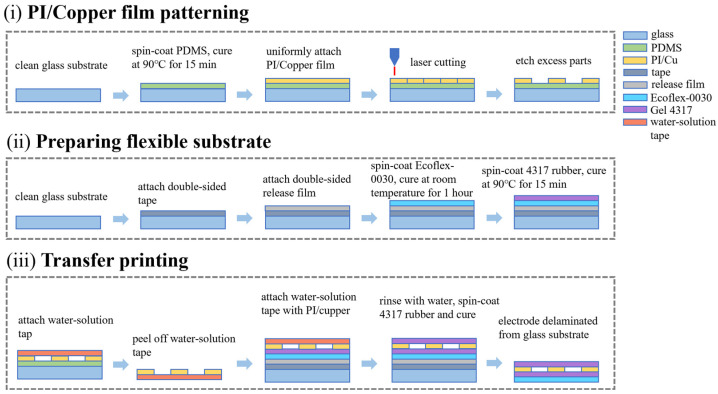
Fabrication of the flexible copper electrode array: (**i**) patterning of the PI/Cu film, involving PDMS sacrificial layer coating, PI/Cu film attachment, laser micromachining, and etching of excess parts; (**ii**) preparation of the flexible substrate, including the attachment of double-sided tape and release film, followed by the sequential spin-coating and curing of Ecoflex-0030 and Gel 4317; (**iii**) transfer printing process, illustrating the retrieval of patterned electrodes using water-soluble tape, transfer to the flexible substrate, and final encapsulation.

The fabrication of the flexible copper electrode array was optimized through a high-precision transfer printing strategy, as illustrated in Figure 4. This process is categorized into three primary stages, each incorporating specific measures to ensure structural integrity and high performance.

In the first stage (Figure 4i), a PDMS sacrificial layer was spin-coated onto a glass substrate before attaching the PI/Cu film. The low surface energy of PDMS is a critical measure that facilitates the damage-free retrieval of the delicate serpentine patterns in subsequent steps. The sensing electrodes were then defined via UV laser micromachining, a technique chosen for its high-resolution and maskless processing capabilities, which significantly reduces manufacturing costs compared to traditional photolithography. The preparation of the flexible substrate (Figure 4ii) involved the sequential spin-coating of Ecoflex-0030 and Gel 4317 rubber. This bilayer configuration strategically combines the excellent mechanical durability of Ecoflex with the superior skin-adhesion and breathability of Gel 4317, creating a compliant base that mimics the modulus of human skin. The final integration was achieved through water-soluble tape-assisted transfer printing (Figure 4iii). The use of water-soluble tape as a temporary carrier ensures that the electrodes can be precisely aligned onto the flexible substrate; furthermore, the complete dissolution of the tape backing in deionized water leaves no sticky residue on the electrode surface, thereby maintaining a clean sensing interface for high-quality signal acquisition.

**Figure 5:**
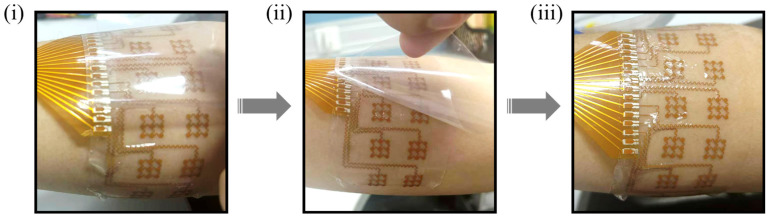
Wearing procedure of the flexible copper electrode array, demonstrating the steps from peeling off the protective film to conformal attachment on the forearm. (**i**) Initial placement. (**ii**) Peeling process. (**iii**) Conformal contact.

The practical advantages of this integrated design are further demonstrated in the wearing procedure (Figure 5). The flexible copper electrode array is designed as a standalone, all-in-one system that can be effortlessly peeled from the release film on the glass carrier (Figure 5ii). This integrated architecture eliminates the need for complex multi-step assembly during use, making the device highly portable and intuitive for end-users. As shown in Figure 5iii, the “peel-and-stick” nature of the array allows for rapid, conformal attachment to the forearm without additional adhesives, providing a convenient and user-friendly tool for daily muscle activity monitoring and home-based rehabilitation training.

### 3.3. Characterization of the Flexible Copper Electrode Array

**Figure 6:**
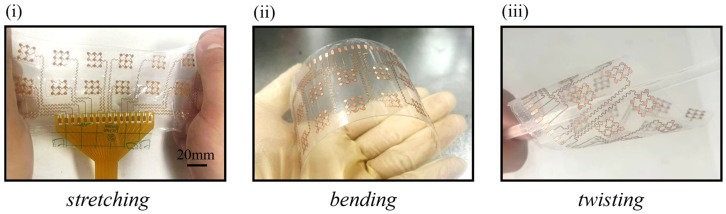
Demonstration of the flexible copper electrode array under various mechanical deformations. (**i**) stretching at ~15% strain (scale bar = 20 mm), (**ii**) bending (bending radius ≈ 25 mm), and (**iii**) twisting (twisting radius ≈ 15 mm).

**Figure 7:**
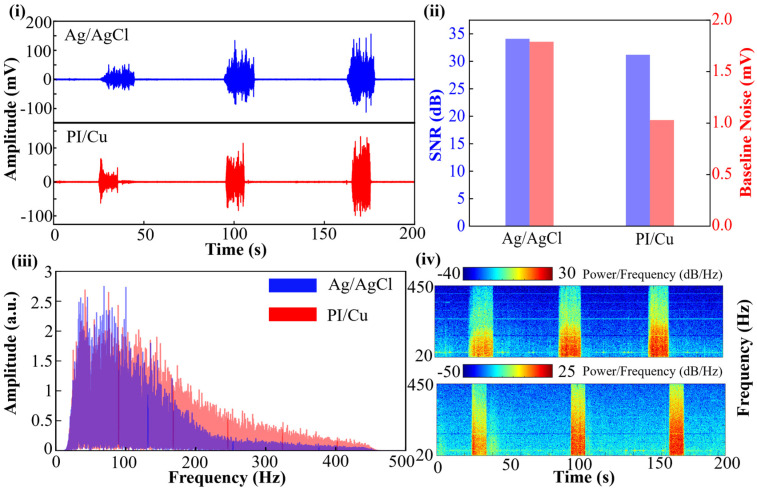
Time-frequency characteristics of the two types of signals. (**i**) Comparison of raw sEMG signals recorded by commercial Ag/AgCl electrodes and the flexible copper electrode array (PI/Cu). (**ii**) Quantitative comparison of the Signal-to-Noise Ratio (SNR) and baseline noise levels between the two types of electrodes. (**iii**) Frequency-domain analysis comparing the amplitude spectra, illustrating the consistency in frequency distribution. (**iv**) Comparison of the time-frequency Power Spectral Density (PSD) analysis for the recorded sEMG signals.

**Figure 8:**
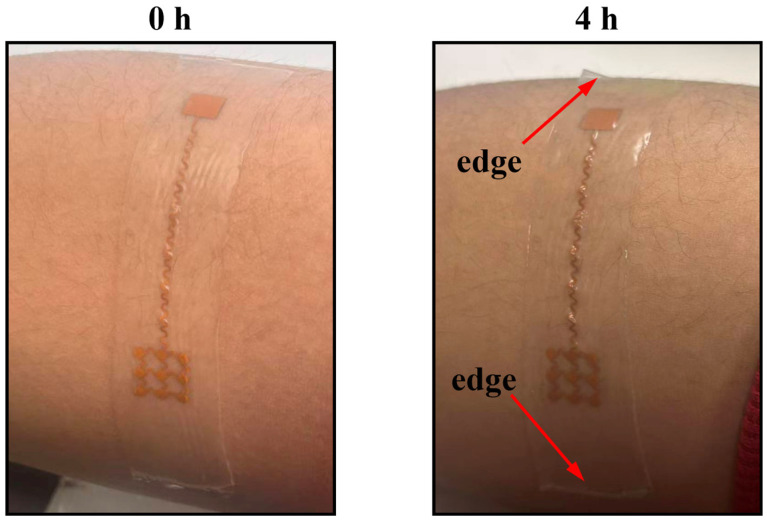
Extended wear stability test of the flexible sensor. Optical images showing the sensor attached to the forearm at 0 h (**left**) and after 4 h of continuous wear during daily activities (**right**). The red arrows indicate that the edges of the sensor remained firmly attached to the skin without any delamination.

To evaluate the practical reliability and signal fidelity of the flexible copper electrode array, we conducted a series of mechanical deformation tests and comparative sEMG acquisition experiments, as summarized in Figure 6, Figure 7 and Figure 8.

The structural integrity of the array under various physiological deformations is a prerequisite for long-term wearable monitoring. As demonstrated in Figure 6, the array exhibits exceptional mechanical robustness, maintaining stable electrical conductivity while subjected to (i) ~15% longitudinal stretching, (ii) bending with a radius of approximately 25 mm, and (iii) twisting with a radius of approximately 15 mm. This high degree of flexibility is primarily attributed to the optimized serpentine interconnects and the thin-film PI/Cu architecture, which allow the device to conformally adapt to the dynamic topography of the human forearm without mechanical failure or delamination.

To quantitatively validate electromechanical stability, a single-channel PI/Cu trace was tested. The end-to-end resistance measured ~1.4 Ω at rest and remained stable at a sub-ohm level (~0.6 Ω) under a 10% tensile strain. This confirms that the serpentine geometry and underlying PI backbone effectively prevent copper micro-cracking, ensuring reliable signal pathways during dynamic skin deformations.

The signal acquisition capability of the proposed array (PI/Cu) was directly compared with commercial pre-gelled Ag/AgCl electrodes. Figure 7i displays the raw sEMG signals captured simultaneously from the same muscle group during three consecutive contraction-relaxation cycles. The temporal waveforms of the PI/Cu electrodes show high synchronization with the Ag/AgCl electrodes, clearly capturing the burst patterns of muscle activity with nearly identical onset and offset timings.

For a more rigorous evaluation, the Signal-to-Noise Ratio (SNR) and baseline noise levels were quantified in Figure 7ii. The SNR was calculated using the following equation:

(1) $$SNR=20{log}_{10}\frac{{RMS}_{signal}}{{RMS}_{noise}},$$

where ${RMS}_{signal}$ and ${RMS}_{noise}$ represent the root mean square (RMS) amplitudes of the sEMG signal during voluntary muscle contraction and the resting baseline state, respectively. Baseline noise was quantified by evaluating the peak-to-peak amplitude during the relaxation periods. To ensure statistical reliability, these metrics were averaged across five independent contraction-relaxation cycles.

Although the SNR of the PI/Cu electrodes (~31 dB) is slightly lower than that of the commercial Ag/AgCl electrodes (~34 dB), it remains well within the high-quality range for reliable gesture decoding. Notably, the baseline noise of the PI/Cu electrodes is controlled at approximately 1.0 mV, which is even lower than that of the commercial electrodes (~1.8 mV). This superior baseline stability suggests that the self-adhesive Gel 4317 encapsulation provides an excellent skin–electrode interface, effectively suppressing ambient noise and motion interference.

To further evaluate the practical adhesion performance of the Gel 4317 layer, an extended continuous wear test was conducted. As shown in Figure 8, the sensor was attached to the forearm of a subject performing routine daily activities. After 4 h of continuous wear, the device maintained conformal contact with the skin. Notably, no edge delamination or macroscopic peeling was observed (indicated by the red arrows), confirming the robust and long-lasting tackiness of the interface under dynamic motion and mild perspiration.

The frequency-domain characteristics further confirm the absence of signal attenuation. The amplitude-frequency spectra in Figure 7iii illustrate that the energy distribution of the PI/Cu electrodes highly overlaps with that of the Ag/AgCl electrodes across the critical 20–450 Hz bandwidth. Furthermore, the time-frequency Power Spectral Density (PSD) analysis in Figure 7iv demonstrates that the intensity and distribution of signal power in both time and frequency axes are remarkably consistent.

**Figure 9:**
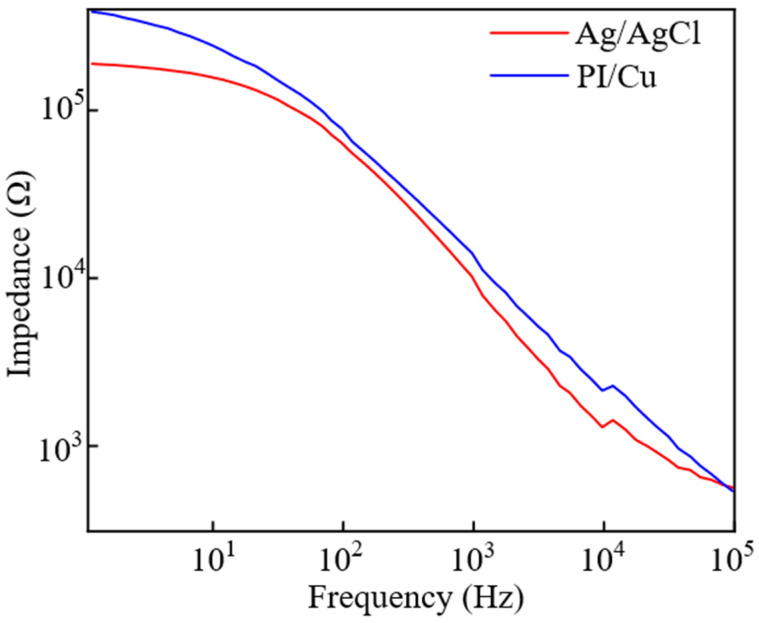
Electrochemical impedance spectra comparing the flexible PI/Cu array and commercial Ag/AgCl electrodes.

To quantitatively evaluate the interface quality, electrochemical impedance was measured from 1 Hz to 100 kHz (Figure 9). The PI/Cu array exhibits an impedance-frequency behavior closely paralleling the commercial pre-gelled Ag/AgCl electrode. Although its impedance is slightly higher at low frequencies—a typical characteristic of gel-free dry electrodes—the PI/Cu array achieves a low impedance of approximately 10^4^ Ω at the standard reference frequency of 1 kHz, highly comparable to the Ag/AgCl control. Furthermore, the curves nearly converge at higher frequencies (>10 kHz), confirming that the conformal flexible design successfully provides a reliable, low-impedance interface for high-fidelity sEMG acquisition.

In conclusion, these characterization results demonstrate that the flexible copper electrode array achieves signal quality comparable to commercial Ag/AgCl electrodes. There is no significant signal attenuation or spectral distortion across the major sEMG frequency bands, proving that our low-cost, flexible copper-based system is a viable and high-performance alternative to traditional rigid sensing technologies.

### 3.4. Application of the Flexible Copper Electrode Array

**Figure 10:**
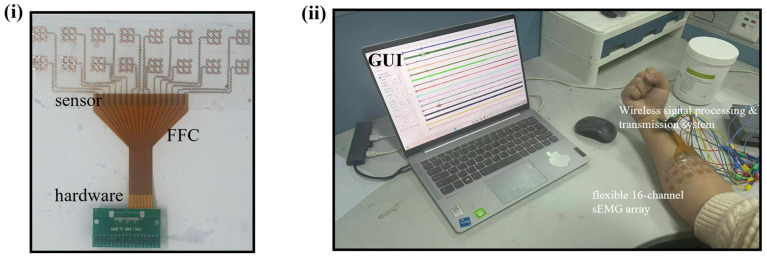
Schematic of the experimental setup and system integration. (**i**) Schematic showing the interconnection between the flexible array, FFC, and acquisition hardware. (**ii**) Experimental scene illustrating the real-time wireless signal acquisition process with the sensor attached to the forearm.

To further validate the practical sensing performance and spatial selectivity of the flexible copper electrode array, we conducted a proof-of-concept multi-channel sEMG acquisition experiment. The preliminary test involved one healthy subject (two males, ages 25; one female, age 26) with varying forearm dimensions. Prior to sensor attachment, the target skin area on the forearm was prepared by wiping with 75% medical alcohol to remove oils and dead skin cells, ensuring an optimal skin–electrode interface. The experimental setup and system integration are illustrated in Figure 10. The flexible array was securely attached to the forearm and interconnected with the customized wireless hardware via a FFC. The captured 16-channel signals were transmitted wirelessly and visualized in real-time on a customized graphical user interface (GUI).

**Figure 11:**
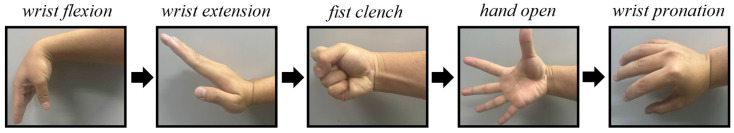
Five representative hand gestures performed during the experiment: wrist flexion, wrist extension, fist clench, hand open, and wrist pronation.

**Figure 12:**
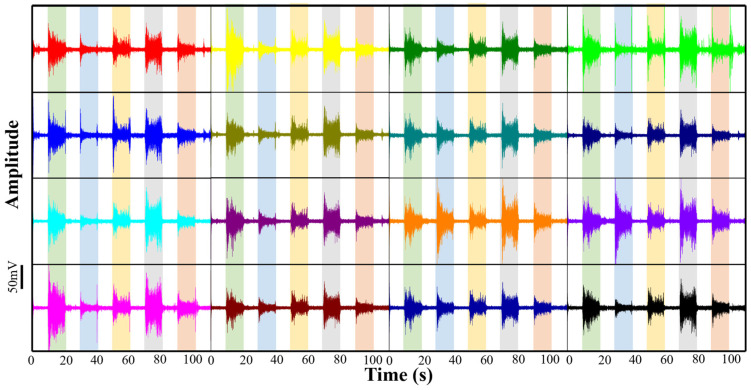
Synchronized 16-channel raw sEMG signals recorded across different gesture cycles (indicated by colored shaded regions), with each waveform color corresponding to a distinct channel, demonstrating the spatial activation diversity of the array.

During the experiment, the subjects were instructed to perform the five distinct hand gestures sequentially, as shown in Figure 11: (1) wrist flexion, (2) wrist extension, (3) fist clench, (4) hand open, and (5) wrist pronation. Each gesture was maintained for approximately 10 s to ensure stable signal capture, followed by a brief relaxation period. To verify the reproducibility and stability of the system, this entire sequence of gestures was repeated five times independently. The synchronized raw sEMG signals from all 16 channels, exhibiting highly consistent activation patterns across all three subjects, are displayed in Figure 12. This figure presents the most representative and typical continuous recording block among the repetitions.

As demonstrated in the time-domain waveforms (Figure 12), the flexible copper electrode array exhibits exceptional sensitivity and spatial specificity across all test scenarios. Upon the initiation of each gesture, distinct “burst” patterns are observed across the array, characterized by high signal-to-noise ratios and minimal baseline drift. Crucially, the activation intensity varies significantly among the 16 channels depending on the specific gesture performed. This spatial specificity is quantitatively demonstrated by evaluating RMS amplitudes across channels. For instance, during wrist flexion (green shaded regions), flexor-associated channels (e.g., Ch1) showed dominant activation with an RMS amplitude of approximately 20 mV, while extensor-associated channels (e.g., Ch 8) remained low (RMS < 3 mV). Conversely, during wrist extension (blue shaded regions), this spatial amplitude pattern inverted completely, with extensor channels peaking at ~18 mV. Meanwhile, the fist clench gesture (yellow regions), requiring multi-muscle co-contraction, triggered a global response with an average multi-channel RMS exceeding 15 mV. These distinct quantitative variations confirm the array’s capability for precise spatiotemporal mapping.

The stability of the 10 s continuous acquisition period for each gesture further confirms the robustness of the skin–electrode interface. Despite the significant muscle bulging and skin deformation associated with these diverse movements, the array maintained conformal contact without generating significant motion artifacts. These results provide strong evidence that the developed 2 × 8 flexible copper electrode array can effectively capture the complex spatiotemporal activation patterns of forearm muscle groups.

## 4. Discussion

The developed flexible copper electrode array demonstrates a strategic balance between high-density sensing performance and cost-effective manufacturing. A primary advantage is the utilization of industrial-standard polyimide-backed copper (PI/Cu) laminates combined with a maskless UV laser micromachining process. This approach eliminates the requirement for expensive noble metals and complex photolithography, providing a scalable route for the mass production of high-density sEMG interfaces.

Mechanically, the structural design featuring serpentine interconnects with circular intersections ensures superior conformal contact with the complex topography of the forearm. These circular nodes serve a dual function: they effectively redistribute mechanical stress during limb deformation and maximize the effective skin–electrode contact area. The increased contact area is critical for achieving low interfacial impedance and high-fidelity signal transduction. Furthermore, the integrated “peel-and-stick” architecture ensures that the sensor is standalone and easy to apply, facilitating stable sEMG acquisition without the need for additional fixation or external adhesives.

From a signal perspective, the 16-channel configuration provides a distributed spatiotemporal dataset that overcomes the information scarcity of traditional sparse electrodes. The array successfully captures distinct muscle activation patterns for various gestures with a signal-to-noise ratio (SNR) comparable to commercial Ag/AgCl electrodes. The integration of a low-power wireless module further supports its potential for long-term, untethered neuromuscular monitoring.

Despite the demonstrated proof-of-concept performance, this study serves primarily as an initial laboratory validation, and several crucial limitations must be addressed before clinical or commercial translation. First, since the array is designed as a disposable sensor for short-term use, initial copper oxidation is functionally negligible. However, future long-term iterations will require standard protective coatings (e.g., OSP) and formal skin biocompatibility assessments. Second, the small subject cohort limits the generalizability of the findings across diverse populations. Third, while our PI/Cu array performs well, broader comparisons with other emerging flexible electrode technologies (e.g., conductive polymers) are necessary to define its competitive edge. Lastly, although we emphasize decoding potential, this work did not implement formal gesture-classification algorithms. Future research will integrate deep-learning frameworks to evaluate actual HMI performance, ultimately advancing this prototype toward a reliable clinical tool.

## 5. Conclusions

In summary, this study developed a cost-effective, high-density flexible copper electrode array for 16-channel sEMG acquisition using standard PI/Cu laminates and a facile UV laser micromachining process. The optimized structural design, featuring serpentine interconnects with circular intersections, ensures superior mechanical robustness and conformal contact with the complex topography of the forearm. Physical and electrical characterizations demonstrate that the array maintains high-fidelity signal quality comparable to commercial Ag/AgCl electrodes (SNR > 30 dB) even under significant mechanical deformations. Furthermore, the integrated wireless system enables the real-time spatiotemporal mapping of forearm muscle activation patterns across diverse hand gestures. This high-performance sensing platform offers a scalable and reliable hardware solution for advanced human–machine interfaces and personalized rehabilitation monitoring, with broad potential for clinical translation in neuromuscular assessment and post-stroke motor recovery.

## Data Availability

The data that support the findings of this study are available from the corresponding author upon reasonable request. The data are not publicly available due to privacy restrictions.
